# Dissecting the Species-Specific Virome in *Culicoides* of Thrace

**DOI:** 10.3389/fmicb.2022.802577

**Published:** 2022-03-07

**Authors:** Konstantinos Konstantinidis, Maria Bampali, Michael de Courcy Williams, Nikolas Dovrolis, Elisavet Gatzidou, Pavlos Papazilakis, Andreas Nearchou, Stavroula Veletza, Ioannis Karakasiliotis

**Affiliations:** ^1^Department of Medicine, Laboratory of Biology, Democritus University of Thrace, Alexandroupolis, Greece; ^2^Evrofarma S.A., Didymoteicho, Greece; ^3^Geotechno Ygeionomiki O.E, Xanthi, Greece

**Keywords:** *Culicoides*, arboviruses, emerging infectious diseases, vectors, virome analysis, metagenomics, Thrace, Greece

## Abstract

Biting midges (*Culicoides*) are vectors of arboviruses of both veterinary and medical importance. The surge of emerging and reemerging vector-borne diseases and their expansion in geographical areas affected by climate change has increased the importance of understanding their capacity to contribute to novel and emerging infectious diseases. The study of *Culicoides* virome is the first step in the assessment of this potential. In this study, we analyzed the RNA virome of 10 *Culicoides* species within the geographical area of Thrace in the southeastern part of Europe, a crossing point between Asia and Europe and important path of various arboviruses, utilizing the Ion Torrent next-generation sequencing (NGS) platform and a custom bioinformatics pipeline based on TRINITY assembler and alignment algorithms. The analysis of the RNA virome of 10 *Culicoides* species resulted in the identification of the genomic signatures of 14 novel RNA viruses, including three fully assembled viruses and four segmented viruses with at least one segment fully assembled, most of which were significantly divergent from previously identified related viruses from the *Solemoviridae*, *Phasmaviridae*, *Phenuiviridae*, *Reoviridae*, *Chuviridae*, *Partitiviridae*, *Orthomyxoviridae*, *Rhabdoviridae*, and *Flaviviridae* families. Each *Culicoides* species carried a species-specific set of viruses, some of which are related to viruses from other insect vectors in the same area, contributing to the idea of a virus-carrier web within the ecosystem. The identified viruses not only expand our current knowledge on the virome of *Culicoides* but also set the basis of the genetic diversity of such viruses in the area of southeastern Europe. Furthermore, our study highlights that such metagenomic approaches should include as many species as possible of the local virus-carrier web that interact and share the virome of a geographical area.

## Introduction

Analyses of arthropod-borne viruses (arboviruses) have spurred the interest of the scientific community. Epidemics caused by yellow fever virus (Cracknell Daniels et al., 2021), Zika virus (Estévez-Herrera et al., 2021), dengue virus (Mayer et al., 2017), West Nile virus (Petersen, 2019), Rift Valley virus (Gregor et al., 2021), chikungunya virus (Mayer et al., 2017), equine encephalitis virus (Lecollinet et al., 2020), lumpy skin disease virus (Klement et al., 2020), and bluetongue virus (BTV) (Saminathan et al., 2020) have constituted a great burden on public health and animal husbandry worldwide over the past few years. Climate change, inducing variations in rainfall, humidity, and wind patterns (Whitehorn and Yacoub, 2019), accompanied by globalization and urbanization phenomena, the alteration of natural habitats, and agricultural structures (Miles et al., 2019), all can affect arthropod reproduction, development, distribution, and feeding behavior. Parallel changes in arthropod ecology may dramatically influence arthropod-borne virus proliferation and transmission.

Among arthropod vectors that include mainly mosquitoes (Culicidae), ticks (Ixodidae), sand flies (Psychodidae, Phlebotominae), black flies (Simuliidae), and biting midges (Ceratopogonidae), the biting midges have received less attention regarding their ability to host or carry viruses, despite the fact that they are the most abundant hematophagous insects worldwide (Sick et al., 2019). The genus *Culicoides* encompasses the most important virus vectors in the family Ceratopogonidae and consists of more than 1,300 identified species (Borkent and Dominiak, 2020) flourishing between moderate climate areas to the tropics. *Culicoides* females feed on vertebrates, with preferences either toward a single species or a wide range of animals (Scholtz, 2018). Most *Culicoides* present crepuscular and nocturnal activity, whereas a few are active mostly during the day time. While they fly mostly in close proximity to their breeding and feeding sites, strong winds may carry them for hundreds of miles affecting their epidemiological importance.

*Culicoides* are of both medical and veterinary importance not only because of their numerical abundance and severity of biting activity but because they carry, either actively or mechanically, a wide range of pathogens, including bacteria, viruses, protozoa, and nematodes (Borkent, 2004; Žiegytė et al., 2021). *Culicoides* species are well known for their role in the emergence and spread of Schmallenberg virus (Rasmussen et al., 2012) (SBV) and BTV (Saminathan et al., 2020), mainly affecting ruminants (cattle, sheep, goats, etc.), and the emergence of Oropouche virus (Sakkas et al., 2018) (OROV) responsible for the Oropouche fever in humans. Most of the viruses transmitted by *Culicoides* are members of the Reoviridae [BTV, epizootic hemorrhagic disease virus, and African horse sickness virus (AHSV)], Rhabdoviridae (bovine ephemeral fever virus), and Peribunyaviridae family [SBV, OROV, and Akabane virus (AKAV)] (Elbers et al., 2015). The geographical distribution of these viruses correlates with the distribution of the respective vector species (Sick et al., 2019). Changes in climate conditions and intensified trade (Elbers et al., 2015) have assisted in the geographic spread of vectors into regions previously naive to viruses such as BTV (Purse et al., 2008) and SBV (Endalew et al., 2019).

An important aspect in the ecology of the viruses vectored by arthropods such as *Culicoides* is the vector specificity and competence. Viruses such as BTV are vectored by multiple species in a specific area (Foxi et al., 2016; Duan et al., 2021). Virus adaptation may determine vector specificity and competence (van Gennip et al., 2019). On the other hand, several *Culicoides* species host or carry multiple viruses. *Culicoides imicola* is a known vector for AKAV, BTV, AHSV, and equine encephalosis virus (Leta et al., 2019), whereas *Culicoides brevitarsis* in Australia presents a similarly wide vector capacity (Tay et al., 2016). The interactions among multiple hosts and viruses in a specific region form a rather complicated network, the elucidation of which necessitates using holistic approaches in the identification of novel virus–host relationships.

Identification of novel viruses in known vectors has been especially highlighted during the coronavirus pandemic as the virus most related to SARS-CoV-2 (severe acute respiratory syndrome coronavirus 2) was identified in virome screenings in bats several years before its emergence in the same geographic area (Menachery et al., 2015; Corman et al., 2018; Morens et al., 2020). Despite the importance of *Culicoides* as vectors, very few studies have been done regarding the analysis of their virome (Temmam et al., 2015, 2016; Modha et al., 2019; Kobayashi et al., 2020; Liu et al., 2020). Metagenomic approaches have successfully yielded various novel and previously known viruses in analyses of single species of *Culicoides impunctatus* populations in Scotland (Modha et al., 2019), *C. imicola* in Senegal (Temmam et al., 2016), and *Culicoides arakawae* in Japan (Kobayashi et al., 2020). On the other hand, mixed populations of *Culicoides* species have been screened for viruses in efforts directed toward developing a more global understanding of the *Culicoides* virome (Temmam et al., 2015; Liu et al., 2020).

During the emergence of a new vector-borne disease, it is important to link the pathogen to its corresponding vector species, in order to promptly access the relevant epidemiological parameters and apply control measures. This is especially important in *Culicoides* as most species have distinct ecological and ethological characteristics, and control measures may vary greatly. Furthermore, biological control using the identified viruses against a certain *Culicoides* species may also be considered a possibility. In the context of this study, a holistic approach is attempted by simultaneously identifying the virome of 10 field-collected *Culicoides* species present in the geographic area of Thrace, Greece, at the southeastern part of Europe, an important path of various arboviruses between the continents of Asia and Europe. In contrast to previous attempts that analyzed either one species or mixtures of species of a taxon (Temmam et al., 2015, 2016; Modha et al., 2019; Kobayashi et al., 2020; Liu et al., 2020), our approach aimed to dissect the local *Culicoides* virome of Thrace and attribute a species-specific virome to all the identified species of *Culicoides* in the area during a collection period. Our methodology was based on the total RNA sequencing of 10 single-species pools followed by a custom bioinformatics pipeline, aiming to investigate, assemble, and phylogenetically characterize the virome of the examined *Culicoides* samples.

## Results

**FIGURE 1:**
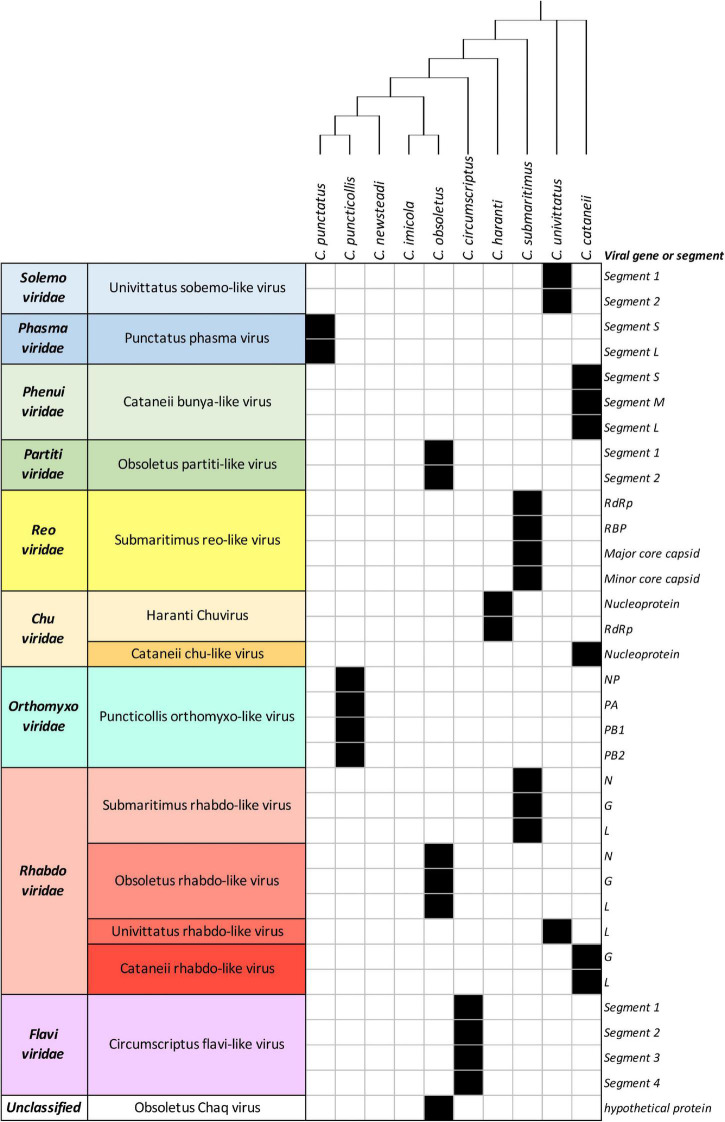
List of detected viruses within *Culicoides* samples. Black filled squares indicate presence of the corresponding viral genes or segments.

The identification of the virome of 10 *Culicoides* species was conducted upon 10 separate pools of *Culicoides* biting midges, which were field-collected from various sites in Thrace, Greece (Supplementary Figure 1), using Centers for Disease Control and Prevention (CDC) light traps. After sorting individuals by morphological identification, with subsequent validation of identifications using DNA barcoding, we analyzed the total RNA virome of 10 *Culicoides* single-species homogenous pools of 10 individuals each. All field-collected *Culicoides* samples underwent a total RNA-Seq protocol based on the Ion Torrent sequencing platform followed by three repetitive assembly runs of the output next-generation sequencing (NGS) data due to the stochastic nature of TRINITY assembler program. The results of Ion torrent sequencing and TRINITY assembly are summarized in Supplementary Tables 1, 2. We were able to identify and assemble 14 RNA viruses (Supplementary Tables 3, 4), all of which were novel virus species to only limited phylogenetic relationship to previously known viruses. In total, all 10 *Culicoides* species showcased a wide range of viruses, which corresponded to 9 viral families: *Solemoviridae*, *Phasmaviridae*, *Phenuiviridae*, *Reoviridae*, *Chuviridae*, *Partitiviridae*, *Orthomyxoviridae*, *Rhabdoviridae*, *Flaviviridae*, and one marked as unclassified at the family level (Figure 1).

All of the examined *Culicoides* species presented a diverse profile among the detected viruses, with *Culicoides obsoletus* and *Culicoides cataneii* being the most potent carriers in our study, carrying three different viruses each. *Culicoides submaritimus* and *Culicoides univittatus* harbored two different viruses each, whereas the majority of the tested field-collected samples (*Culicoides puncticollis*, *Culicoides punctatus*, *Culicoides circumscriptus*, *Culicoides haranti*) possessed a single virus species each. Notably, *Culicoides newsteadi* and *C. imicola* did not yield any viral sequences in the context of this study, despite both being notorious virus vectors. The virome of each *Culicoides* sample is described analytically per examined species in the following paragraphs.

### Culicoides punctatus

**FIGURE 2:**
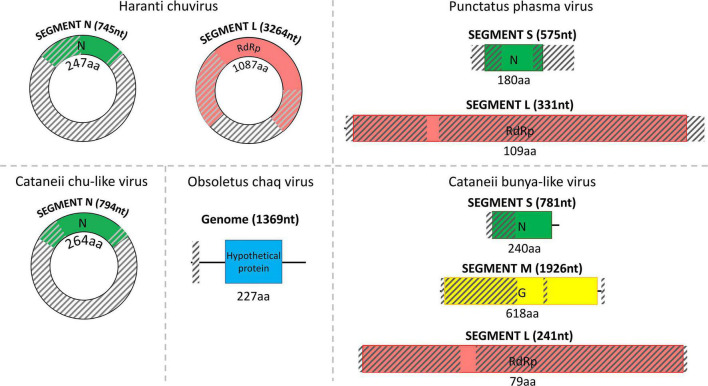
Structure of the detected chu/chu-like viruses, phasma virus, bunya-like virus, and uncharacterized Chaq virus separated by dashed lines. The total length of each assembled viral sequence is indicated in nucleotides between parentheses (nt), whereas the corresponding encoded protein length is shown below each colorful rectangular region in amino acids (aa). Translation of all viral genomic sequences was done by ExPASy Translate online tool. The diagonally shaded regions upon each viral genomic sequence depict areas that could not be successfully assembled, and their lengths were estimated after MAFFt alignment against the most closely related viral nucleotide sequences.

*C. punctatus* harbored a *punctatus* phasma virus, member of the *Phasmaviridae* viral family, which was also partially assembled. Members of the family *Phasmaviridae* typically comprise three diverse genomic segments, namely, the nucleocapsid (segment S), glycoprotein (segment M), and RdRp (segment L) (Figure 2). However, the M segment could not be detected here, whereas the rest of the segments were recovered successfully from the examined samples of *C. punctatus*. The assembled N segment of *punctatus* phasma virus returned the nucleocapsid protein of Wuhan mosquito virus 1 (YP_009305133.1) as best overall BLASTp hit, whereas the assembled L segment matched the corresponding RdRp of *Aedes* phasmavirus (QOI91399.1) after a BLASTp search.

### Culicoides submaritimus

**FIGURE 3:**
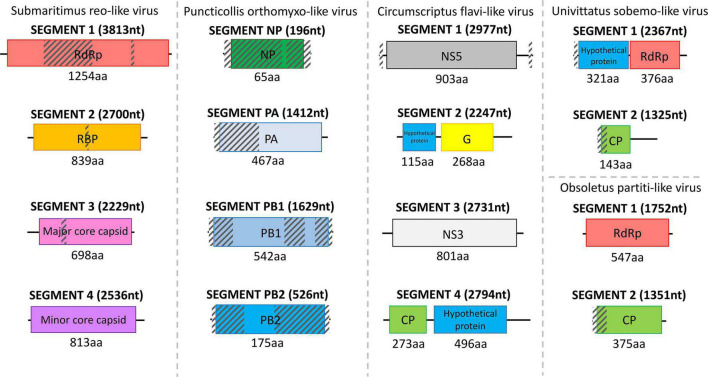
Structure of the detected reo-like virus, orthomyxo-like virus, flavi-like virus, sobemo-like virus, and partiti-like virus separated by dashed lines. The length of each assembled viral sequence is indicated in nucleotides between parentheses (nt), whereas the corresponding encoded protein length is shown below each colorful rectangular region in amino acids (aa). Translation of all viral genomic sequences was done by ExPASy Translate online tool. The diagonally shaded regions upon each viral genomic sequence depict areas that could not be successfully assembled, and their lengths were estimated after MAFFt alignment against the most closely related viral nucleotide sequences.

Two viruses were detected within *C. submaritimus* belonging to the families *Reoviridae* and *Rhabdoviridae*, namely, submaritimus reo-like virus and submaritimus rhabdo-like virus, respectively. Four segments of submaritimus reo-like virus were successfully identified, constituting the RNA-dependent RNA-polymerase (RdRp), the RNA-binding protein (RBP), the major core capsid, and the minor core capsid (Figure 3). Only the latter was assembled completely, whereas the rest of the segments exhibited minor fragmentation. The RdRp segment of submaritimus reo-like virus showed the highest identity to the putative RdRp of Atrato reo-like virus (QHA33828). Similarly, the RBP and major core capsid segments of submaritimus reo-like virus matched the corresponding RBP (QHA33825) and major core capsid (QHA33826) segments of the aforementioned Atrato reo-like virus. The fully assembled minor core capsid segment of submaritimus reo-like virus displayed the highest coverage and aa identity compared with the rest of the detected segments, returning the minor core capsid of Hubei reo-like virus 11 (APG79054) as top BLASTp hit.

**FIGURE 4:**
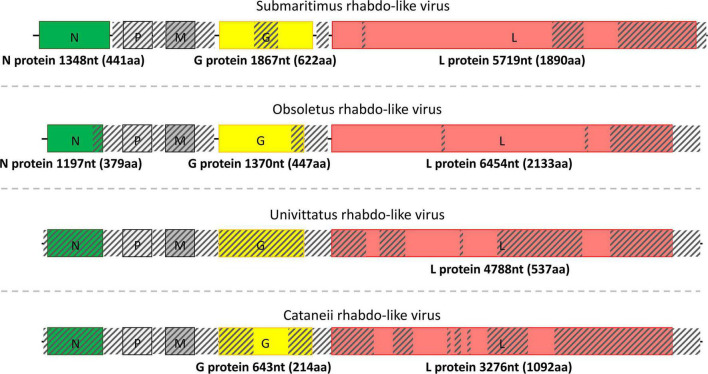
Structure of the detected rhabdo-like viruses separated by dashed lines. The length of each assembled viral sequence is indicated in nucleotides between parentheses (nt), whereas the corresponding encoded protein length is shown below each colorful rectangular region in amino acids (aa). Translation of all viral genomic sequences was done by ExPASy Translate online tool. The diagonally shaded regions upon each viral genomic sequence depict areas that could not be successfully assembled, and their lengths were estimated after MAFFt alignment against the most closely related viral nucleotide sequences.

Moreover, submaritimus rhabdo-like virus was retrieved with its nearly complete nucleoprotein (N protein), as well as the partially assembled glycoprotein (G protein) and RdRp (L protein), whereas matrix and phosphoprotein transcripts could not be recovered (Figure 4). The best BLASTp match of submaritimus rhabdo-like virus nucleoprotein (NP) was the hypothetical protein of Wuhan mosquito virus 9 (QTW97821.1). Glycoprotein of submaritimus rhabdo-like virus BLASTp aligned against the glycoprotein of Sanxia Water Strider Virus 5 (YP_009289351.1), whereas the assembled RdRp yielded the RNA-dependent RNA polymerase of Guadeloupe *Culex* rhabdovirus (QEM39120.1) as top BLASTp hit.

### Culicoides puncticollis

Only 1 virus was obtained from *C. puncticollis* species, which carried a puncticollis orthomyxo-like virus, member of the *Orthomyxoviridae* viral family, consisting of four partially assembled genomic segments, specifically one NP and three discrete polymerases (PA, PB1, PB2) (Figure 3). Despite the small length of the assembled puncticollis orthomyxo-like virus NP segment, BLASTp reported the nucleocapsid protein of Jingshan fly virus 1 (APG77879.1) as best match. The assembled PA segment was highly similar to the PA polymerase of *Aedes alboannulatus* orthomyxo-like virus (ASA47422.1). The remaining PB1 and PB2 polymerases matched PB1 of Wuhan mosquito virus 3 (AJG39091.1) and PB2 of *A. alboannulatus* orthomyxo-like virus (ASA47421.1), respectively.

### Culicoides univittatus

Field-collected samples of *C. univittatus* showed the species was infected with two different viruses, a univittatus sobemo-like virus and a univittatus rhabdo-like virus, belonging to the families *Solemoviridae* and *Rhabdoviridae*, respectively. *Solemoviridae* and sobemo-like viruses typically consist of two discrete genomic segments, each one encoding two proteins (Figure 3). Similarly, in this study, the first segment of univittatus sobemo-like virus encoded two separate proteins. The protein encoded first in this segment was identified as a putative protein, similar to the hypothetical protein of *Vespa velutina* RNA virus 3 (ATY36114.1) after a BLASTp search, whereas the second protein product of the same segment showed the highest identity to the RdRp of Atrato sobemo-like virus 1 (QHA33896.1). Nevertheless, the second segment of univittatus sobemo-like virus encoded only one protein noted as capsid. The assembled capsid of univittatus sobemo-like virus was closely related to the putative coat protein of Atrato sobemo-like virus 2 (QHA33889.1) according to BLASTp results. Univittatus rhabdo-like virus was also detected and recovered successfully from *C. univittatus*. However, it was assembled only partially as most of its genes were missing except for the encoded RdRp (Figure 4). The assembled RdRp of univittatus rhabdo-like virus showed the highest identity to the RNA-dependent RNA polymerase of *Culex pseudovishnui* rhabdo-like virus (BBQ04832.1) via BLASTp.

### Culicoides obsoletus

*C. obsoletus* was one of the most potent virus carriers of the examined species, carrying two viruses belonging to *Partitiviridae* and *Rhabdoviridae* families and one unclassified virus. Obsoletus partiti-like virus, obsoletus rhabdo-like virus, and an unclassified obsoletus Chaq virus were detected within the field-collected samples of *C. obsoletus*. Like all partitiviruses, obsoletus partiti-like virus had a bipartite genome, with each segment encoding the RdRp and capsid proteins of the virus, respectively, which were both assembled successfully (Figure 3). The RdRp segment of obsoletus partiti-like virus yielded the corresponding RdRp of Hubei diptera virus 17 (YP_009337870.1) as top BLASTp hit, whereas the assembled capsid segment BLASTp matched the putative capsid protein of Atrato partiti-like virus 2 (QHA33903.1). A rhabdo-like virus (obsoletus rhabdo-like virus) was also identified in this study, possessing monopartite linear genomic structure and sequentially transcribing the NP, phosphoprotein, matrix, glycoprotein, and RdRp genes as usual for members of the family Rhabdoviridae. Three of the transcribed genes were almost fully assembled, but the phosphoprotein and matrix genes could not be retrieved (Figure 4). The NP of obsoletus rhabdo-like virus showed the highest identity to the putative NP of *Culex* rhabdo-like virus (AXQ04770.1), whereas BLASTp of the assembled glycoprotein returned the glycoprotein of Ohlsdorf virus (ATG83561.1) as a top hit. The RdRp of obsoletus rhabdo-like virus BLASTp matched the polymerase-associated protein of Ohlsdorf virus (ATG83562.1). Notably, *C. obsoletus* carried an unclassified obsoletus Chaq virus, whose closest relative was the corresponding ORF of Chaq-like virus (QMI58124.1) after BLASTp search (Figure 2).

### Culicoides cataneii

Similarly, *C. cataneii* harbored three viruses, namely, *cataneii* bunya-like virus, *cataneii* chu-like virus, and *cataneii* rhabdo-like virus, which are members of the families *Phenuiviridae*, *Chuviridae*, and *Rhabdoviridae*, respectively. The *cataneii* bunya-like virus was successfully recovered from the field-collected samples of *C. cataneii*, but its genomic segments were only partially assembled. The *cataneii* bunya-like virus consisted of three separate segments, encoding the corresponding nucleocapsid (segment S), glycoprotein (segment M), and RdRp (segment L) proteins (Figure 2). The assembled segment S of *cataneii* bunya-like virus yielded the nucleocapsid of *Austropotamobius* brown spot virus (QCO84581.1) as top BLASTp hit, whereas the assembled glycoprotein BLASTp matched the glycoprotein of Dar es Salaam virus TZ-189 (QDF82061.1). A BLASTp of the RdRp of *cataneii* bunya-like virus returned the L protein of Blacklegged tick phlebovirus 1 (ANT80544.1) as top hit. A member of the recently described family *Chuviridae*, *cataneii* chu-like virus was detected in this study, but only the NP gene of which could be assembled partially (Figure 2). The NP of *cataneii* chu-like virus BLASTp aligned against the putative NP of Atrato chu-like virus 1 (QHA33915.1). Another member of the family *Rhabdoviridae* was found within *C. cataneii*, named *cataneii* rhabdo-like virus. The glycoprotein and RdRp genes of *cataneii* rhabdo-like virus were recovered successfully and assembled partially, whereas the rest of the viral genes could not be retrieved (Figure 4). The glycoprotein of Riverside virus 1 (YP_009552819.1) BLASTp matched the *cataneii* rhabdo-like virus glycoprotein. The large protein (RdRp) of Riverside virus 1 (AMJ52373.1) was the top BLASTp hit of *cataneii* rhabdo-like virus RdRp.

### Culicoides circumscriptus

Interestingly, *C. circumscriptus* carried *circumscriptus* flavi-like virus, which resembled members of the family *Flaviviridae*. Although *circumscriptus* flavi-like virus characteristics have not been described fully, six viral genes were recovered successfully and assembled completely from the field-collected samples of *C. circumscriptus* (Figure 3). Notably, both the fully assembled sequences for capsid and glycoprotein encoded an extra protein product, in one case after and in the other case before these transcribed genes. More specifically, the assembled capsid of *circumscriptus* flavi-like virus showed the highest identity to the putative capsid protein of Wuhan aphid virus 2 (QDF44112.1), whereas the protein product following capsid transcription BLASTp matched the hypothetical protein of Wuhan aphid virus 1 (BBV14760.1). Similarly, an extra protein product was found before glycoprotein transcription highly similar to the hypothetical protein of Wuhan aphid virus 1 (YP_009179380.1), whereas the main glycoprotein product itself was closer to the putative glycoprotein of Wuhan aphid virus 2 (QDF44110.1) via BLASTp searches. The remaining two assembled genes displayed high similarities with the corresponding NS3 and NS5 transcribed genes of flavi-like viruses. The NS3 of *circumscriptus* flavi-like virus retrieved NS3-like protein of soybean thrips virus 4 (QPZ88419.1) as the top BLASTp hit. Furthermore, its assembled NS5 showed the highest identity to the NS5-like protein of Wuhan aphid virus 1 (QPZ88370.1).

### Culicoides haranti

*C. haranti* also presented interesting findings in the tested field-collected samples as they harbored haranti chuvirus, a member of the recently described family *Chuviridae*. With little prior knowledge regarding the genomic structure and other characteristics of this viral family, two genes of haranti chuvirus were recovered successfully but assembled only partially (Figure 2). The assembled NP of haranti chuvirus BLASTp matched the nucleocapsid protein of Coleopteran chu-related virus (QMP82322.1). Lastly, the RdRp of haranti chuvirus showed the highest identity to the RNA-dependent RNA polymerase of Hubei chuvirus-like virus 3 (YP_009337089.1).

### Phylogenetic Analysis of Novel Viruses

**FIGURE 5:**
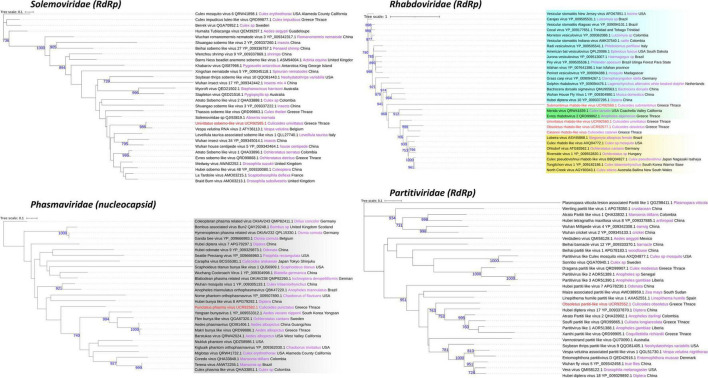
*Solemoviridae*, *Phasmaviridae*, *Rhabdoviridae*, and *Partitiviridae* family phylogenetic trees of the identified viruses in this study (red text). Phylogenetic analysis was performed according to the protein indicated between the parentheses after each viral family name using its amino acid sequence. FastME minimum evolution substitution model was utilized as part of the NGPhylogeny.fr methodology. All of the presented phylogenetic trees were rooted according to the outgroup rooting method. Bootstrap values (blue text) were obtained from 1,000 bootstrap replicates, and only those greater than 700 are displayed at the start of each node. Host and country origin information of homologous viruses was also extracted, if applicable, depicted here in purple and black text, respectively. Viruses with known genus taxonomy have also been highlighted accordingly. More specifically, *Orthophasmavirus* genus is colored gray in the *Phasmaviridae* family, whereas *Sigmavirus*, *Ohlsrhavirus*, and *Merhavirus* genera are displayed with cyan, yellow, and green gradients, respectively, in the *Rhabdoviridae* family.

**FIGURE 6:**
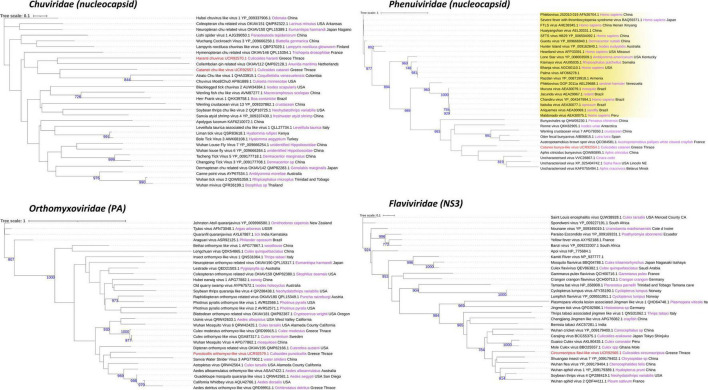
*Chuviridae*, *Orthomyxoviridae*, *Phenuiviridae*, and *Flaviviridae* family phylogenetic trees of the identified viruses in this study (red text). Phylogenetic analysis was performed according to the protein indicated between the parentheses after each viral family name using its amino acid sequence. FastME minimum evolution substitution model was utilized as part of the NGPhylogeny.fr methodology. All of the presented phylogenetic trees were rooted according to the outgroup rooting method. Bootstrap values (blue text) were obtained from 1,000 bootstrap replicates, and only those greater than 700 are displayed at the start of each node. Host and country origin information of homologous viruses was also extracted, if applicable, depicted here in purple and black text, respectively. Viruses with known genus taxonomy have also been highlighted accordingly. More specifically, *Phlebovirus* genus is colored yellow in the *Phenuiviridae* family.

**FIGURE 7:**
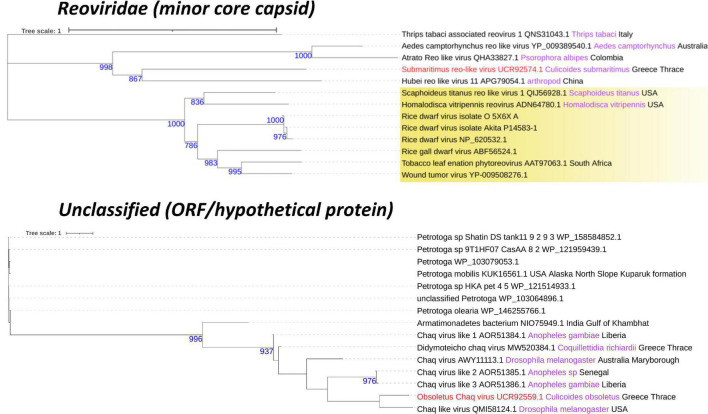
*Reoviridae* and unclassified family phylogenetic trees of the identified viruses in this study (red text). Phylogenetic analysis was performed according to the protein indicated between the parentheses after each viral family name using its amino acid sequence. FastME minimum evolution substitution model was utilized as part of the NGPhylogeny.fr methodology. All of the presented phylogenetic trees were rooted according to the outgroup rooting method. Bootstrap values (blue text) were obtained from 1,000 bootstrap replicates, and only those greater than 700 are displayed at the start of each node. Host and country origin information of homologous viruses was also extracted, if applicable, depicted here in purple and black text, respectively. Viruses with known genus taxonomy have also been highlighted accordingly. More specifically, *Phytoreovirus* genus is colored yellow in the *Reoviridae* family.

An assessment of the phylogeny of the identified new viruses revealed both local and global links with viruses of other invertebrates but also vertebrates. Univittatus sobemo-like virus belonged to a cluster of viruses identified mainly in insects such as mosquitoes (Figure 5). Within the same cluster, a related virus in *Culex theileri* had been identified before in the area of Thrace (Thassos sobemo-like virus) (Figure 5). The only vertebrate virus in the cluster was an uncharacterized *Sobemovirus* identified in the bird *Abrornis inornate* (*Solemoviridae* sp. QJI53819.1) (Figure 5). Punctatus phasma virus was clustered within the characterized genus of *Orthophasmaviruses* containing the related Carapha virus identified in *C. arakawae* in Shinjuku, Tokyo, Japan (Figure 5). Interestingly, a distinct subgroup that comprised mostly mosquito viruses within the genus contained Makri bunya-like virus identified in *Aedes albopictus* by our group in the same region (deposited in NCBI GenBank, QRD99886.1) (Figure 5). Another member of the order of *Bunyavirales*, *cataneii* bunya-like virus clustered together with other members of the *Phenuiviridae* family and very close to a cluster containing important human phleboviruses such as Chandiru virus and Maldonado virus (Figure 6). Submaritimus reo-like virus was very similar to other unclassified insect reoviruses closely related to *Phytoreovirus* genus of plant viruses within the Sedoreovirinae subfamily (Figure 7). Four *Culicoides* species harbored a rhabdo-like virus of the Alpharhabdovirinae subfamily. Submaritimus rhabdo-like virus formed a branch between *Sigmavirus* and *Merhavirus* cluster as exemplified by *Bactrocera dorsalis* sigmavirus and Merida virus, respectively (Figure 5). Univittatus rhabdo-like virus was mostly related to the *Merhavirus* cluster and interestingly related to another virus (Evros rhabdovirus 2) identified in the same region in *Anopheles algeriensis* mosquitoes (Figure 5). Obsoletus rhabdo-like virus and *cataneii* rhabdo-like virus formed their own cluster between Merhaviruses and Ohlsrhaviruses (Figure 5). Obsoletus partiti-like virus was clustered only with unclassified Partitiviruses, intriguingly, in a subcluster that contained previously identified Partitiviruses of mosquitoes (*Culiseta longiareolata* and *Coquillettidia richiardii*) in the same area (Figure 5). Haranti chuvirus and *cataneii* chu-like virus were both clustered closely within the novel *Chuviridae* family (Figure 6). Puncticollis orthomyxo-like virus was clustered with Sanxia water strider virus 3 and other Orthomyxoviruses belonging to the *Quaranjavirus* genus (Figure 6). Circumscriptus flavi-like virus was closely related to insect-specific viruses but phylogenetically distant from human flaviviruses such as yellow fever virus and Saint Louis encephalitis virus (Figure 6). Obsoletus Chaq virus was another virus within an unclassified family of Chaq viruses that have been proposed to be satellite viruses of other viruses (Shi et al., 2018). Didymoteicho Chaq virus detected in *C. richiardii* mosquitoes in the area of Thrace was also closely related to Obsoletus Chaq virus (Figure 7).

In terms of phylogeography, the viruses identified in this study did not cluster according to area, country, or continent. Although in some cases such as in *Solemoviridae*, *Rhabdoviridae*, *Phasmaviridae*, and *Partitiviridae* families, the novel viruses reported in this study were closely related to viruses previously identified in mosquitoes in the area of Thrace, Greece.

## Materials and Methods

### *Culicoides* Collection and Identification

Adult *Culicoides* were field-collected using CDC light traps with Photo Cell and Air Gate (BioQuip Products, Inc., United States) (Supplementary Table 5). Collection points spanned across the areas of Eastern Macedonia and Thrace in Greece (Supplementary Figure 1) during a period of intensified *Culicoides* activity; April–October 2019. Post collection, the samples were stored and delivered on dry ice. *Culicoides* specimens were examined over a bed of crushed ice to maintain their condition at all times, both during sample sorting and during species identification. Good-quality intact individuals were stored at −80°C prior to RNA and DNA extraction. Female *Culicoides* were identified using external morphological features (Mathieu et al., 2012).

### Species Identification Through Cytochrome C Oxidase Subunit 1 Barcoding

*Culicoides* were homogenized using pellet pestle (Eppendorf, Germany), and total RNA was extracted by TRIzol reagent (Thermo Fisher Scientific, United States) according to the manufacturer’s protocol. As verification of the morphological identification, DNA barcoding was done using standard cytochrome c oxidase subunit 1 (COI) polymerase chain reaction (PCR) and Sanger sequencing. One microgram of the RNA extract was reverse-transcribed at 42°C for 60 min, using M-MLV reverse transcriptase (Promega, United States) according to the manufacturer and COI_R primer (5′-AAAAATTTTAATTCCAGTTGGAACAGC-3′). Universal primers COI_F (5′-GGATTTGGAAATTGATTAGTTCCTT-3′) and COI_R were used to amplify a 600-bp PCR product. The PCR reaction mixture contained 0.25 × GC buffer, 1.5 mM MgCl_2_, 1 mM dNTPs mix, 0.2 μM of each primer, 1.5 U KAPA Taq DNA polymerase (Kapa Biosystems, United States), and 1 μL of cDNA. The thermal profile of the PCR included 40 cycles of denaturation at 95°C for 30 s, annealing at 50°C for 45 s, and elongation at 65°C for 1 min, and a final elongation step at 65°C for 7 min. PCR products were purified using the NucleoSpin Gel and PCR Clean-up purification kit (Macherey-Nagel, Germany). Sanger sequencing was performed on the PCR product, and the resulting sequence was analyzed using the Barcode of Life Data System V4 platform (Ratnasingham and Hebert, 2007) and tested vs. local COI sequences submitted in the NCBI Genbank database (Supplementary Table 6).

### Total RNA Next-Generation Sequencing

*Culicoides* biting midges were separated into homogenous single-species pools of 10 well-preserved individuals each, from various collection sites within the Thrace region (two individuals from every area presented in Supplementary Figure 1), and total RNA was extracted by TRIzol reagent (Thermo Fisher Scientific, United States) and pellet pestle (Eppendorf, Germany) according to the manufacturer’s protocol. The quality of the RNA preparation was assessed using LabChip GX Touch 24 (PerkinElmer, United States) capillary electrophoresis. Whole transcriptome libraries were prepared from 500 ng of total RNA, using the Ion Total RNA-Seq v2 Core Kit (#4479789, Thermo Fisher Scientific, United States) according to the manufacturer’s instructions. In brief, the RNA library preparation involved RNA fragmentation, adapter ligation, reverse transcription, and 14 cycles of PCR amplification using Ion Xpress™ RNA-Seq Barcode 1–16 Kit (#4475485, Thermo Fisher Scientific, United States). Quantification of the library was performed using Qubit Fluorometer high-sensitivity kit (Thermo Fisher Scientific, United States), and its median size was determined in LabChip GX Touch 24 (PerkinElmer, United States). The libraries were loaded on an Ion 540 chip (yielding approximately 80 million reads using the 200-bp chemistry), using the automated Ion Chef System (Thermo Fisher Scientific, United States), and sequencing was carried out on an Ion GeneStudio S5, Ion Torrent sequencer (Thermo Fisher Scientific, United States) with the NGS results summarized in Supplementary Table 1. The datasets presented in this study can be found in the SRA database of NCBI^1^ identified by their accession numbers as shown in Supplementary Table 7.

### Viral Genome Assembly

Following the NGS procedure, raw sequences from the sampled homogenous *Culicoides* single-species pools of 10 individuals each (Supplementary Table 1) were used as input for *de novo* assembly using Trinity (Grabherr et al., 2011) (v2.8.5.). Trinity assembler, based on the de Bruijn graph algorithm, produces contigs (set of overlapping DNA segments), which represent alternate transcripts of genes, treating sequences with structural changes (mutations and indels) as isoforms of the same gene. The whole process was performed via three distinct modules, namely, Inchworm, Chrysalis, and Butterfly, each responsible for creating the assemblies of transcripts, clustering them and optimizing the de Bruijn graphs, respectively. Because of the non-deterministic/probabilistic nature of the algorithm, each sample/pool was submitted to three Trinity assembly runs, using the default program parameters, thus maximizing the possibilities of revealing *bona fide* full-length viral sequences. The output data of TRINITY assembly are summarized in Supplementary Table 2. The generated and assembled contigs of all Trinity runs were aligned against the non-redundant (nr) protein database via BLASTx (Altschul et al., 1990) using taxonomic search restriction on viridae (taxid:10239) and annotated by their top BLASTx hit. In addition, sequences corresponding to the same top BLASTx hit were fed into CAP3 online tool (Huang and Madan, 1999) with default parameters for the creation of scaffolds by overlapping contigs, aiming to maximize viral genome assembly efficiency. Lastly, alignment algorithms [Burrows-Wheeler Aligner (Li and Durbin, 2009), MAFFt (Katoh et al., 2002)] and Integrated Genomics Viewer (Robinson et al., 2011) program were utilized in order to fine tune and validate the assembled viral sequences before submitting them to the NCBI GenBank database. All assembled viral sequences of this study can be accessed online by their accession numbers (MZ771201- MZ771234) (Supplementary Table 3).

### Phylogenetic Analysis

For the phylogenetic analysis of *Culicoides* species, the COI sequences of the respective species (Supplementary Table 5) were input to the *NGPhylogeny.fr* website (Lemoine et al., 2019) running a custom workflow with successive stages of multiple-sequence alignment by MAFFT (Katoh, 2002), alignment refinement by BMGE (Criscuolo and Gribaldo, 2010), and phylogenetic reconstruction via FastME substitution model (Lefort et al., 2015), based on balanced minimum evolution for 1,000 bootstrap cycles and, finally, graphical representation of the inferred tree through Newick Display. Tree was exported to iTol (Letunic and Bork, 2019). *Culicoides* phylogeny was verified by previously constructed phylogenetic trees (Morag et al., 2012; Augot et al., 2017).

For the phylogenetic analysis of the assembled viruses, the amino acid sequence of the RNA-dependent RNA polymerase or RdRp (also characterized as segment L or L protein) was used for the construction of the phylogenetic trees. In case the RdRp segment was not assembled, nucleocapsid (segment S or N) or glycoprotein (segment M or G) amino acid sequence was used. The length of the input sequence varied, depending on the length of the assembled contigs. The respective virus amino acid sequence obtained from ExPASy Translate online tool (Gasteiger et al., 2003) according to the standard genetic code was input to BLASTp against the nr protein sequence database of NCBI, and hits with not less than 25% coverage and 30% identity were selected. Host and geographic origin data were extracted through the NCBI tool *Entrez-direct* (Kans, 2021) by using a custom in-house bash script (available in github).^2^ All previously selected sequences were fed to the *NGPhylogeny.fr* website (Lemoine et al., 2019) for the elucidation of phylogenetic relationships, running a custom workflow with successive stages of multiple sequence alignment by MAFFT (Katoh, 2002); alignment refinement by BMGE (Criscuolo and Gribaldo, 2010); phylogenetic reconstruction via FastME substitution model (Lefort et al., 2015), based on balanced minimum evolution for 1,000 bootstrap cycles; and finally, graphical representation of the inferred trees through Newick Display. Trees were rooted using the outgroup rooting method and were exported to iTol (Letunic and Bork, 2019).

## Discussion

The recurrent outbreaks of BTV (Foxi et al., 2016; Saminathan et al., 2020; Duan et al., 2021), the discovery of SBV in Europe (Carpenter et al., 2013; Endalew et al., 2019), and the wide distribution of OROV in Southern America (McGregor et al., 2021) have highlighted the importance of understanding *Culicoides* ecology and biology. As climate change alters vector ecology and distribution, vector-borne pathogens find new paths in naive territories (Purse et al., 2008). Despite the great economic (Gethmann et al., 2020) and public health impact of the viruses vectored by *Culicoides*, knowledge relating the ecology and epidemiology of *Culicoides*-specific viruses is lagging behind that of viruses transmitted by other major arthropod vectors. Analysis of the virome in insect vectors, such as mosquitoes, has given clues on their ability to harbor a large variety of viruses (Cook et al., 2013; Huhtamo et al., 2014; Atoni et al., 2019). Such endeavors have revealed the potential of vectors to harbor viruses closely related to human pathogens such as flaviviruses (Cook et al., 2013; Huhtamo et al., 2014; Atoni et al., 2019). Either transmitted to another animal species or as being insect-specific, hundreds of novel arboviruses have been identified through RNA metagenomics (Öhlund et al., 2019a; Sanborn et al., 2019; Shi et al., 2019; Ramírez et al., 2020; de Almeida et al., 2021; Thannesberger et al., 2021). Although we know very little about these novel viruses their impact on vector competence in relation to previously known pathogenic arboviruses has been extensively studied (Öhlund et al., 2019b). It is believed that the insect core virome, a virome that shows relative stability between individuals of the same species (Shi et al., 2019), presents similarities to the human microbiome, which has been shown to affect the ability of pathogens to establish growth and induce disease. However, most of the reports have shown negative or inconclusive results on the effect of insect specific viruses on important human or animal pathogens (Kent et al., 2010; Bolling et al., 2012; Pereira et al., 2018; Talavera et al., 2018; Koh et al., 2021; Utarini et al., 2021). With mosquito vectors, coinfection with the insect-specific virus *Culex* flavivirus (Bolling et al., 2012; Talavera et al., 2018) did not alter competence in relation to Rift Valley fever phlebovirus and showed variable results with respect to vector competence for West Nile virus (Kent et al., 2010). Similarly, with mosquitoes of the genus *Aedes*, an infection with the insect specific Palm Creek virus did not affect competence of the same mosquitoes toward Zika and chikungunya virus (Koh et al., 2021). Although these observations confute the hypothesis, previous experiments on bacterial species that affect vector competence have shown considerable effect with *Wolbachia* species (Pereira et al., 2018; Utarini et al., 2021). As vector-borne diseases are soaring worldwide, there is an increased effort for the identification of vector competence altering agents for disease control. The continuous identification and characterization process of novel viromes are essential for the isolation of competence altering viral agents but also for the understanding of emerging pathogen potential.

In our study, we aimed to identify novel viruses in field populations of a range of *Culicoides* species in the area of Thrace, northeast Greece, collected during the monitoring period April–October 2019. This region is of great zoogeographic importance as it bridges three distinct biogeographic realms, and it constitutes an area that is a major route of various animal and human pathogens from Asia to Europe [e.g., BTV (Purse et al., 2008), lumpy skin disease virus (Tasioudi et al., 2016), sheep pox virus (Mangana et al., 2008), *Plasmodium vivax* (Danis et al., 2013), and West Nile virus (Erdem et al., 2014)], the majority of which are vector-borne pathogens. An RNA-seq–based methodology for the metagenomic analysis of transcriptomes of all *Culicoides* species collected in the region of Thrace in 2019 was used to elucidate their core virome in a holistic but also species-specific approach for this period. From the morphological evaluation of the collected samples, the individuals were divided into 10 species. Aiming to identify the core virome of these species that has been shown before to be stable in other vectors such as mosquitoes (Shi et al., 2019, 2020), we processed 10 well-preserved (dry ice) specimens from each species as a homogenous pool.

Using the Ion Total RNA-Seq v2 Core Kit for total RNA sequencing and the Ion Torrent S5 sequencer, we prepared libraries of pools of 10 representative individuals for each species. After TRINITY assembly of all possible contigs, we identified sequences belonging to 14 distinct and novel viruses, members of the *Solemoviridae*, *Phasmaviridae*, *Phenuiviridae*, *Partitiviridae*, *Reoviridae*, *Chuviridae*, *Rhabdoviridae*, and *Flaviviridae*, as well as sequences of a virus similar to the unclassified Chaq virus. Interestingly, every virus was identified in only one *Culicoides* species, whereas several species of biting midges harbored multiple viruses, in line with the notion of a species-specific virome that has been described before for mosquitoes (Shi et al., 2019). However, more individuals are required for thorough identification of the virome of each species as several viruses may be less abundant or are present in low viral load. Previous reports on virus metagenomics of *Culicoides* showed a similar composition in terms of viral families, although the reports either focused on a single species (Modha et al., 2019) or the genus as a whole (Liu et al., 2020) (i.e., pools of different species).

Partial (8) and near-complete or complete (6) genomes corresponding to species of the *Solemoviridae*, *Phasmaviridae*, *Phenuiviridae*, *Partitiviridae*, *Reoviridae*, *Chuviridae*, *Rhabdoviridae*, and *Flaviviridae* virus families were identified (Figures 2–4). The phylogenetic analysis of these viruses gave insights into their relationships with other previously identified viruses in the area but also worldwide. Most of the viruses identified in the virome of *Culicoides* biting midges from Thrace clustered together with previously identified—mostly arthropod-specific, viruses identified in aphids, flies (including mosquitoes), and ticks. Although viruses from mosquitoes seem to populate most of the phylogenetic clusters, the bias resulting from the extensive virome analysis in mosquitoes should be taken into consideration. Viruses similar to puncticollis othomyxo-like virus have been identified before only in mosquito species of the genera *Culex* (Batson et al., 2021) and *Aedes* (Shi et al., 2017; Batson et al., 2021; Figure 6). Obsoletus rhabdo-like virus, submaritimus rhabdo-like virus, *cataneii* rhabdo-like virus, and univittatus rhabdo-like virus formed a cluster that encompassed mainly similar viruses detected in mosquito species of the genera *Anopheles*, *Aedes* (including *Ochlerotatus* subgenus) (Reuter et al., 2016; Shahhosseini et al., 2017), and *Culex* (Coffey et al., 2014; Hang et al., 2016; Sadeghi et al., 2018; Öncü et al., 2018; Faizah et al., 2020; Batson et al., 2021; Figure 5). The aforementioned *Rhabdoviruses* were phylogenetically related to *Sigmavirus*, *Ohlsrhavirus*, and *Merhavirus* genera coclustering with Evros rhabdovirus 2 and Merida-like virus (Ergünay et al., 2017) identified in the Greek and Turkish part of Thrace region, respectively. Similarly, *cataneii* chu-like virus was related to viruses found previously infecting mosquito species of the genera *Culiseta* (API61889.1) and *Coquillettidia* (QHA33915.1) (Figure 6). Interestingly, obsoletus partiti-like virus, similar to arthropod viruses from China (Shi et al., 2016), Colombia, and Liberia, clustered together with two viruses identified previously by our group in the mosquito species *C. longiareolata* (QRD99865.1) and *C. richiardii* (QRD99905.1) in Thrace (Figure 5). Similarly, univittatus sobemo-like virus was related to Thassos sobemo-like virus identified in *C. theileri* in the same region, although more closely related to *V. velutina* RNA virus 2 identified in the Belgium (Garigliany et al., 2017; Figure 5). This co-occurrence of similar viruses in different vector groups highlights the possibility of interactions occurring among different arbovirus vector species in the area. A recent extensive analysis of *Culicoides* and mosquitoes assessed the potential virome interactions between the two taxa in China (Liu et al., 2020). Comparative analysis highlights the fact that there is a web of viruses and carriers in the ecosystem where hosts/carriers exchange viruses, which eventually evolve to adapt among host species. Occasionally some viruses may “leak” out of the web, infecting vertebrates occurring in close proximity with the vectors.

As very few studies have been conducted on *Culicoides* virome, it is not surprising that only Carapha virus identified in *C. arakawae* in Japan (Kobayashi et al., 2020) was related to a virus from this study (*punctatus* phasma virus) (Figure 5). To be noted, *C. arakawae* and *C. punctatus* were phylogenetically distant within the genus (Morag et al., 2012). Often, insect-specific viruses are closely related to viruses detected or isolated in plants and fungi (Vasilakis and Tesh, 2015; Franco et al., 2021). Hypotheses that attempt to explain such an observation include a central role of the insects in a “one-health” concept of vector-borne diseases of both plants and animals (Dietzgen et al., 2016) or contamination of insect samples with fungi (Cook et al., 2013; Chandler et al., 2015). Studies that include virome characterization after a cell culture on insect cells may support or confute the above hypotheses (Pyke et al., 2021). Submaritimus reo-like virus fell into the same phylogenetic clade with three other insect viruses, namely, *Aedes camptorynhus* reo-like virus, Atrato reo-like virus and Hubei reo-like virus. This cluster formed a distant phylogenetic clade to viruses of the genus *Phytoreovirus* of *Reoviridae* that infect plants such as rice and tobacco as reported previously (Shi et al., 2017; Figure 7).

Members of the uncharacterized group of Chaq viruses and the recently described *Chuviridae* family were identified in three *Culicoides* species, namely, obsoletus Chaq virus, haranti chuvirus, and *cataneii* chu-like virus. Chaq viruses were proposed to be satellite viruses of other viruses, as in its initial identification, Chaq virus contigs were always present together with Galbut virus during viral metagenomics (Shi et al., 2018). In our study, we did not identify any contigs corresponding to Galbut virus or any other related viruses. *Chuviridae* is a family of arthropod viruses often found only in metagenomic studies with variable genomic structure (Dezordi et al., 2020). The close proximity of haranti chuvirus and *cataneii* chu-like virus with Wuchang cockroach virus 3 within the phylogenetic tree and their distance from Tacheng tick virus 5 and Bole tick virus 3 favored a segmented genome structure depiction (type II) as proposed by Li et al. (2015; Figure 6). Moreover, chuviruses have been linked to endogenous viral elements (EVEs) that include either small or larger fragments of insect-specific viruses incorporated into insect genomes (Aguiar et al., 2015; Dezordi et al., 2020). It is possible that viruses represented by a partial fragment or a single segment may actually be EVEs as even sequences of well-established viral families such as *Flaviviridae* have been shown to form EVEs (Lequime and Lambrechts, 2017). However, it should be noted that the vast majority of viruses identified in this study comprised either large contigs or multiple segments (Figures 2–4).

Some of the most important vector-borne viruses that infect humans and animals are members of the family *Flaviviridae*. The family is divided into distinct clusters that contain either vertebrate or invertebrate-specific viruses (de Oliveira Ribeiro et al., 2021). A flavivirus that clustered together with invertebrate-specific viruses was identified in *C. circumscriptus*. In our study, only one virus clustered together with non-arthropod viruses. A bunyavirus similar to *cataneii* bunya-like virus in the past had been identified in the fecal virome of otters in Spain (Bodewes et al., 2014; Figure 6). Within the same tree of *Phlebovirus* (*Phenuiviridae*, *Bunyavirales*), *cataneii* bunya-like virus was closer to a group of viruses from aphids (Zhang et al., 2019), whereas a neighboring cluster contained important human *Phlebovirus* such as Chandiru virus (Palacios et al., 2011) and Maldonado virus (Palacios et al., 2011; Figure 6). The possibility that these viruses are transferred to vertebrates or have the potential to underscores the importance of metagenomic approaches that link viruses with specific species in the same habitat. These data provide an important component in the development of risk assessments of the new pathogens occurring and could be used to screen co-occurring mammal and avian species that act as hosts for blood feeding vectors and the viruses they harbor.

Our work follows a new global approach in the study of *Culicoides* virome, assigning viruses to specific species occurring naturally within the same geographical area. The new viruses identified help us understand the web of virus–host/carrier interactions at the ecosystem level in Thrace. Finally, our study provides a database useful for the wider geographical area of southeastern Europe in the analysis of vector and virus distributions and risk assessment analyses for emerging infectious diseases.

## Data Availability Statement

The datasets presented in this study can be found in online repositories. The names of the repository/repositories and accession number(s) can be found below: https://www.ncbi.nlm.nih.gov/, SRR15194102, SRR15194673, SRR15194737, SRR15194761, SRR15194763, SRR15194765, SRR15194766, SRR15194470, SRR15194764, and SRR15194762.

## Author Contributions

IK designed the study. IK and SV obtained funding for the project. AN and PP conducted fieldwork and collected samples. MC performed morphological species identification. KK, MB, and EG performed experiments. KK and ND performed the analysis. KK, MB, and IK wrote the manuscript with input from all authors. All authors contributed to the article and approved the submitted version.

## Conflict of Interest

PP was employed by the company Evrofarma S.A. AN was employed by the company Geotechno Ygeionomiki O.E. The remaining authors declare that the research was conducted in the absence of any commercial or financial relationships that could be construed as a potential conflict of interest.

## Publisher’s Note

All claims expressed in this article are solely those of the authors and do not necessarily represent those of their affiliated organizations, or those of the publisher, the editors and the reviewers. Any product that may be evaluated in this article, or claim that may be made by its manufacturer, is not guaranteed or endorsed by the publisher.

## Footnotes

1
https://www.ncbi.nlm.nih.gov/sra/

2
https://github.com/konskons11/konskons11/blob/NCBI_HOST-COUNTRY_DATA/NCBI_VIRUS_data.sh
