# Supplementary material for: Dissecting the Species-Specific Virome in Culicoides of Thrace
Source: Front Microbiol. 2022 Mar 7;13:802577. doi: 10.3389/fmicb.2022.802577 (PMC8940260; doi:10.3389/fmicb.2022.802577)
Supplement: Supplementary file 1 [file Data_Sheet_1.docx]

**SUPPLEMENTARY TABLES**

Supplementary Table 1. Summary of the Ion Torrent sequencing output data per examined Culicoides species

| **Examined Species** | **Total number of reads** | **Total length of reads (bp) *** | **Average read length (bp) *** | **Total number of ribosomal reads** |
| --- | --- | --- | --- | --- |
| *Culicoides submaritimus* | 10,892,044 | 1,444,833,960 | 132.65 | 2,279,102 |
| *Culicoides puncticollis* | 8,591,840 | 879,408,040 | 102.35 | 2,306,105 |
| *Culicoides punctatus* | 6,841,815 | 788,228,875 | 115.21 | 2,165,325 |
| *Culicoides obsoletus* | 8,657,213 | 1,011,616,616 | 116.85 | 2,929,777 |
| *Culicoides newsteadi* | 8,909,863 | 956,427,876 | 107.34 | 2,436,390 |
| *Culicoides univittatus* | 9,583,731 | 1,332,632,947 | 139.05 | 2,506,822 |
| *Culicoides imicola* | 8,487,736 | 932,411,313 | 109.85 | 3,846,669 |
| *Culicoides circumscriptus* | 7,990,698 | 902,660,195 | 112.96 | 2,138,815 |
| *Culicoides haranti* | 6,100,232 | 619,211,858 | 101.51 | 1,228,554 |
| *Culicoides cataneii* | 6,658,220 | 673,846,774 | 101.21 | 1,335,499 |

- Read lengths post quality trimming

Supplementary Table 2. Summary of the TRINITY assembly program output data. The depicted arithmetic values correspond to the average of 3 TRINITY assembly runs per examined Culicoides species.

| **Examined Species** | **Total number of sequences** | **Total length of sequences (bp)** | **Average sequence length (bp)** |
| --- | --- | --- | --- |
| *Culicoides submaritimus* | 2,048 | 933,569 | 455.80 |
| *Culicoides puncticollis* | 1,057 | 473,764 | 448.38 |
| *Culicoides punctatus* | 1,146 | 514,779 | 449.10 |
| *Culicoides obsoletus* | 1,341 | 604,724 | 450.77 |
| *Culicoides newsteadi* | 1,178 | 492,306 | 417.95 |
| *Culicoides univittatus* | 1,735 | 809,768 | 466.64 |
| *Culicoides imicola* | 777 | 355,404 | 456.95 |
| *Culicoides circumscriptus* | 1,758 | 848,660 | 482.68 |
| *Culicoides haranti* | 785 | 355,763 | 453.25 |
| *Culicoides cataneii* | 829 | 375,822 | 453.26 |

*Supplementary Table 3. Viruses identified in the study with their NCBI GenBank accession number.*

| Virus | Accession number | Virus | Accession number |
| --- | --- | --- | --- |
| Obsoletus Partiti-like virus | MZ771201 MZ771202 | **Submaritimus Rhabdo-like virus** | MZ771215  MZ771216  MZ771217 |
| Cataneii Bunya-like virus | MZ771203  MZ771204  MZ771205 | **Haranti Chuvirus** | MZ771218  MZ771219 |
| Cataneii Chu-like virus | MZ771206 | **Submaritimus Reo-like virus** | MZ771220  MZ771221  MZ771222  MZ771223 |
| Cataneii Rhabdo-like virus | MZ771207  MZ771208 | **Obsoletus Rhabdo-like virus** | MZ771224  MZ771225  MZ771226 |
| Obsoletus Chaq virus | MZ771209 | **Puncticollis Orthomyxo-like virus** | MZ771227  MZ771228  MZ771229  MZ771230 |
| Univittatus Rhabdo-like virus | MZ771210 | **Punctatus Phasma virus** | MZ771231  MZ771232 |
| Circumscriptus Flavi-like virus | MZ771211  MZ771212  MZ771213  MZ771214 | **Univittatus Sobemo-like virus** | MZ771233  MZ771234 |

Supplementary Table 4. Total number of viral reads and viral abundance per virus from the studied Culicoides samples.

| ***Virus family*** | ***Virus name*** | ***Mapped reads**** | ***Viral abundance***** | ***Mapped reads  average length*** |
| --- | --- | --- | --- | --- |
| *Solemoviridae* | Univittatus sobemo-like virus | 1945 | 203.2 | 149.25 |
| *Phasmaviridae* | Punctatus phasma virus | 87 | 5.0 | 132.79 |
| *Phenuiviridae* | Cataneii bunya-like virus | 309 | 46.4 | 111.4 |
| *Partitiviridae* | Obsoletus partiti-like virus | 7248 | 837.8 | 135.56 |
| *Reoviridae* | Submaritimus reo-like virus | 1913 | 175.7 | 143.36 |
| *Chuviridae* | Haranti Chuvirus | 354 | 58.0 | 106.72 |
|  | Cataneii chu-like virus | 56 | 4.4 | 99.1 |
| *Orthomyxoviridae* | Puncticollis orthomyxo-like virus | 240 | 27.7 | 109.18 |
| *Rhabdoviridae* | Submaritimus rhabdo-like virus | 1524 | 140.6 | 144.42 |
|  | Obsoletus rhabdo-like virus | 1050 | 121.3 | 129.01 |
|  | Univittatus rhabdo-like virus | 122 | 12.7 | 150.57 |
|  | Cataneii rhabdo-like virus | 119 | 17.9 | 112.61 |
| *Flaviviridae* | Circumscriptus flavi-like virus | 4952 | 620.3 | 120.97 |
| *Unclassified* | Obsoletus Chaq virus | 1589 | 183.5 | 125.88 |

* Number of reads after BWA alignment running with default parameters between each assembled viral sequence and the respective NGS sample which was predominantly used for virus assembly.
** Viral abundances calculated by the RSEM software expressed in transcripts per million (TPM).

Supplementary Table 5. Number of individuals collected per Culicoides species and in total (areas are marked on the Supplementary figure)

| **Examined Species** | **Number of individuals** | **Area 1** | **Area 2** | **Area 3** | **Area 4** | **Area 5** |
| --- | --- | --- | --- | --- | --- | --- |
| *Culicoides submaritimus* | 19 | 2 | 3 | 2 | 3 | 9 |
| *Culicoides puncticollis* | 620 | 120 | 65 | 35 | 176 | 224 |
| *Culicoides punctatus* | 412 | 19 | 12 | 6 | 128 | 247 |
| *Culicoides obsoletus* | 312 | 23 | 56 | 12 | 176 | 45 |
| *Culicoides newsteadi* | 664 | 287 | 58 | 78 | 143 | 98 |
| *Culicoides univittatus* | 124 | 16 | 23 | 12 | 34 | 39 |
| *Culicoides imicola* | 104 | 25 | 2 | 2 | 28 | 47 |
| *Culicoides circumscriptus* | 60 | 5 | 12 | 34 | 2 | 7 |
| *Culicoides haranti* | 32 | 7 | 3 | 2 | 8 | 12 |
| *Culicoides cataneii* | 17 | 2 | 3 | 3 | 4 | 5 |
| ***TOTAL*** | **2364** | **505** | **237** | **187** | **702** | **733** |

Supplementary Table 6. NCBI GenBank accession number list of cytochrome c oxidase subunit 1 (COI) sequences used for barcoding.

| **Examined Species** | **GenBank Accession number** |
| --- | --- |
| *Culicoides submaritimus* | MZ695761 |
| *Culicoides puncticollis* | MZ695762 |
| *Culicoides punctatus* | MZ695763 |
| *Culicoides obsoletus* | MZ695764 |
| *Culicoides newsteadi* | MZ695765 |
| *Culicoides univittatus* | MZ695766 |
| *Culicoides imicola* | MZ695767 |
| *Culicoides circumscriptus* | MZ695768 |
| *Culicoides haranti* | MZ695769 |
| *Culicoides cataneii* | MZ695770 |

Supplementary Table 7. Accession number list of the studied Culicoides species after submission to NCBI SRA database.

| **Examined Species** | **SRA Accession number** |
| --- | --- |
| *Culicoides submaritimus* | SRR15194102 |
| *Culicoides puncticollis* | SRR15194470 |
| *Culicoides punctatus* | SRR15194673 |
| *Culicoides obsoletus* | SRR15194737 |
| *Culicoides newsteadi* | SRR15194766 |
| *Culicoides univittatus* | SRR15194765 |
| *Culicoides imicola* | SRR15194764 |
| *Culicoides circumscriptus* | SRR15194763 |
| *Culicoides haranti* | SRR15194762 |
| *Culicoides cataneii* | SRR15194761 |

**SUPPLEMENTARY FIGURE**

**a**

**b**


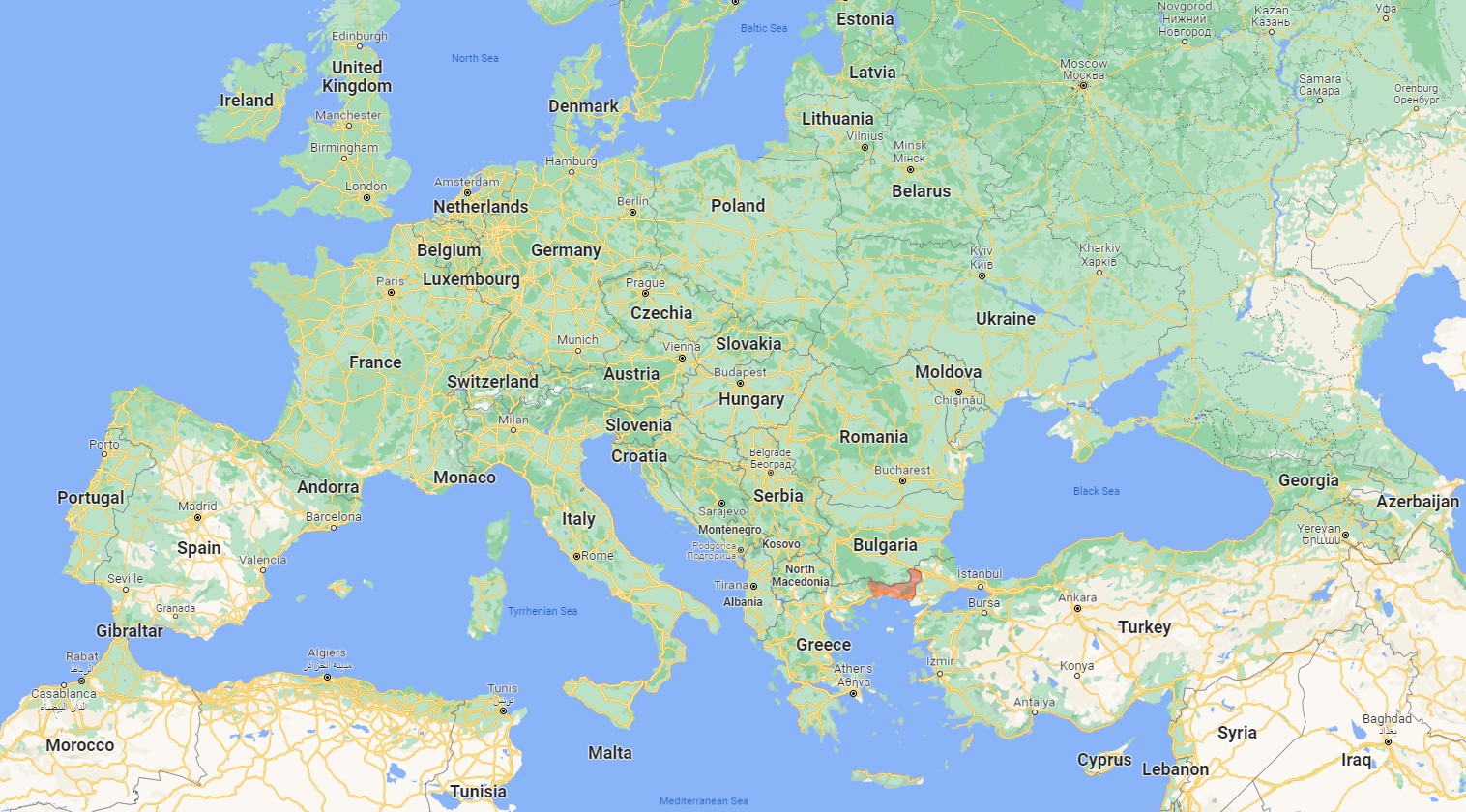


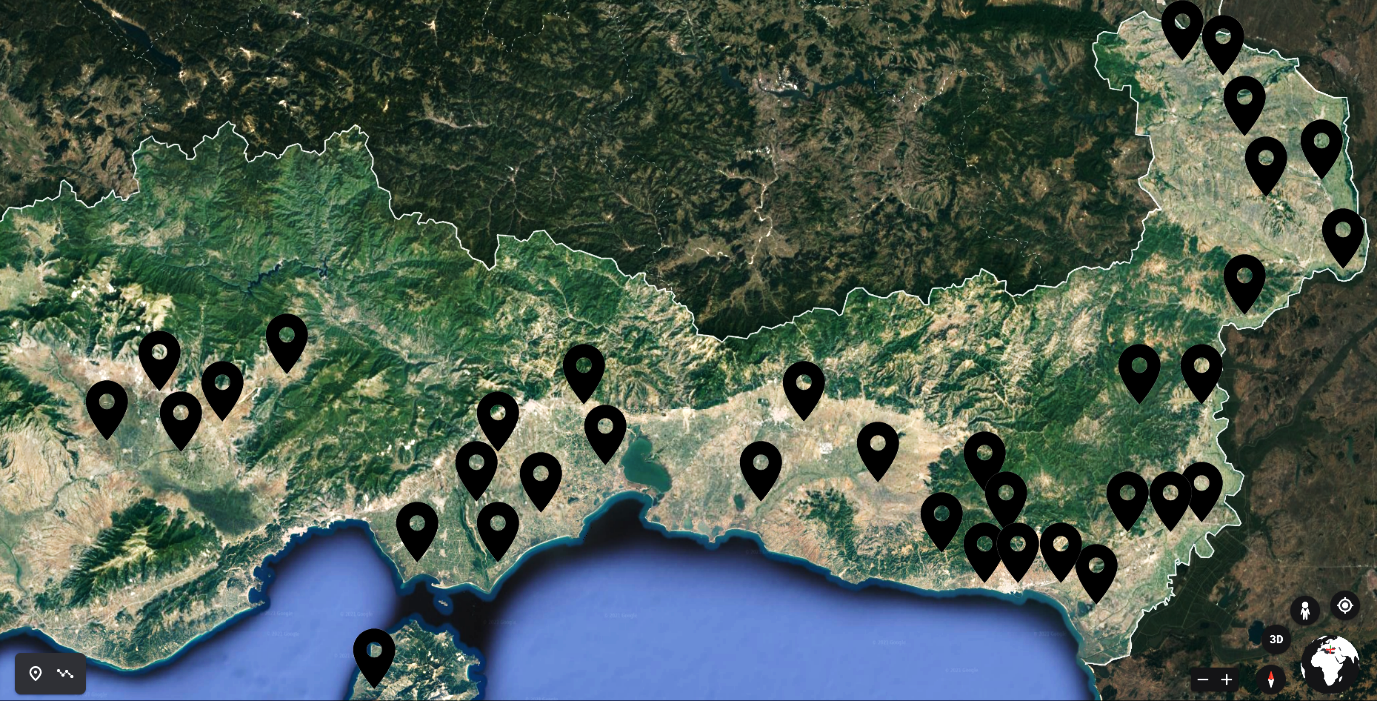


**5**

**4**

**3**

**2**

**1**

*Supplementary Figure 1. a) Geopolitical map including in red shade the region of Thrace, Greece. b) Physical map of the collection sites in the Thrace region of Greece. The collections were organized into 5 smaller geographical areas Drama (1), Xanthi (2), Rhodopi (3), South Evros (4), North Evros (5). The area is bordered to the north with Bulgaria and to the East with Turkey. Location black marks indicate the collection sites.*
